# Treatments for Cannabis Use Disorder across the Lifespan: A Systematic Review

**DOI:** 10.3390/brainsci14030227

**Published:** 2024-02-28

**Authors:** Mohammad Ghafouri, Sabrina Correa da Costa, Ali Zare Dehnavi, Mark S. Gold, Teresa A. Rummans

**Affiliations:** 1 Department of Psychiatry & Psychology, Mayo Clinic, 200 First St. SW, Rochester, MN 55901, USA; ghafouri.mohammad@mayo.edu (M.G.);; 2 Department of Psychiatry, Washington University School of Medicine, St. Louis, MO 63110, USA; drmarksgold@gmail.com; 3 Department of Psychiatry, Mayo Clinic, Jacksonville, FL 32224, USA

**Keywords:** cannabis use disorder, CUD, psychotropic medications, behavioral interventions, substance use disorder

## Abstract

Cannabis use disorder (CUD) is a growing public health concern, with rising prevalence and significant impact on individuals across age groups. This systematic review examines 24 studies investigating pharmacological and non-pharmacological interventions for CUD among adolescents (up to 17), young adults (18–24), and older adults (25–65). Database searches were conducted for randomized controlled trials of CUD interventions reporting outcomes such as cannabis use, abstinence, withdrawal symptoms, and treatment retention. For adolescents, interventions such as contingent rewards and family engagement have shown promise, while young adults benefit from technology-based platforms and peer support. In older adults, pharmacological adjuncts combined with counseling have shown promise in enhancing treatment outcomes. However, optimal treatment combinations remain uncertain, highlighting the need for further research. Addressing CUD requires tailored interventions that acknowledge developmental stages and challenges across the lifespan. Although promising interventions exist, further comparative effectiveness research is needed to delineate the most efficacious approaches.

## 1. Introduction

Cannabis use disorder (CUD) is characterized by problematic patterns of cannabis use leading to clinically significant impairment or distress [1]. The prevalence of CUD has risen in parallel with increasing potency of cannabis products and growing legalization over the past two decades, now affecting nearly 1.5% of the US population [2,3]. CUD is associated with significant health and social costs, including increased psychosis risk, cognitive impairment, poorer mental health, and declines in education and occupation [1]. Recent research continues to reveal additional harms related to CUD. For instance, a study reports higher rates of violence and self-harm among patients with comorbid cannabis use disorders as compared to non-users [4,5]. These behaviors can result in injury and elevated risk of suicide, especially in adolescents and younger adults [6]. This underscores the urgent need to prioritize interventions targeting problematic cannabis use from both a treatment and policy standpoint, aiming to alleviate the associated harms.

Rates of cannabis use and associated disorders demonstrate considerable variability across different age groups [7,8]. For instance, adolescents and young adults have the highest rates of cannabis use, with one study showing that over 35% of 12th graders had reported use within the previous year [7,8]. Consequently, younger demographic groups are more vulnerable to developing CUD, which can have detrimental effects on neurological development, educational attainment, and psychosocial functioning [9,10]. Among adults, it seems that cannabis use peaks during the ages of 18–25, gradually declining thereafter [11]. However, older adults remain susceptible to problematic cannabis use that interferes with occupational, medical, or social domains.

Treatments for CUD have demonstrated relative efficacy, particularly in alleviating withdrawal symptoms, although the effects of existing treatments on relapse prevention and abstinence remain suboptimal. The main modalities of care include pharmacological and psychosocial interventions. Pharmacotherapies such as gabapentin, N-acetylcysteine, and cannabinoid receptor 1 (CB1) agonists have shown promise, while psychosocial approaches, including motivational interviewing (MI), cognitive behavioral therapy (CBT), and contingency management (CM) have demonstrated clinical benefit [12]. Unfortunately, there has been little examination of the relative efficacy of these therapeutic interventions across different age groups.

Given age-specific variability in the patterns and contexts underlying disordered cannabis use and its consequences, interventions tailored to specific developmental stages may confer superior treatment outcomes. Adolescents and young adults frequently initiate cannabis use recreationally and socially, often lacking insights into problematic behaviors. Thus, psychoeducation and motivational enhancement strategies could potentiate other therapies, for instance. Conversely, older adults more commonly use cannabis under the premise of “self-medicating” underlying psychiatric or medical conditions, suggesting that pharmacological interventions may play a more robust role in treatment outcomes; thus, combined strategies targeting both maladaptive cognitions and behaviors besides psychiatric and non-psychiatric comorbidities may be more effective [13].

The comparative effectiveness of current CUD interventions across adolescents, young adults, and older adults remains unknown. Our systematic review aimed to examine the literature with the hopes of bridging this gap in the existing literature by examining the efficacy of pharmacological and non-pharmacological interventions for cannabis use disorder across age groups. If successful, findings from our study will help to establish age-specific evidence-based strategies for the treatment of CUD, besides future directions in clinical research, to improve the assessment and management of this growing public health concern.

## 2. Methods

This systematic review was conducted in accordance with the Preferred Reporting Items for Systematic Reviews and Meta-Analyses (PRISMA) guidelines [14]. The review protocol was registered with PROSPERO (registration number: CRD42024503653).

### 2.1. Search Strategy

A systematic literature search was undertaken to identify studies evaluating pharmacological and non-pharmacological interventions for CUD across adolescent, young adult, and older adult populations. We searched the following electronic databases from 1 January 2013 to 31 December 2023: PubMed, Scopus, PsycINFO, and ClinicalTrials.gov. The search strategies were developed in collaboration with an experienced medical research librarian with expertise in systematic review searching. The search concepts combined terms related to:

(1) CUD and associated terminology (e.g., “cannabis/marijuana abuse”, “cannabis/marijuana dependence”, “cannabis use disorder”) AND (2) pharmacological interventions, e.g., nabilone, bupropion, gabapentin, cannabidiol, psychedelics “OR” (3) non-pharmacological (psychosocial and behavioral) interventions.

The search strategies were customized for each database using applicable controlled vocabularies and search syntax. The full electronic search strategies utilized for all databases are presented in Appendix A file in Supplementary Materials. EndNote X20 reference management software was utilized to collate records retrieved from the literature search (Table 1).

**Eligibility criteria:** Randomized controlled trials (RCTs) that met the following inclusion criteria: (1) participants diagnosed with CUD, heavy cannabis users, treatment-seeking individuals, or subjects screened as “problematic” cannabis users. (2) RCT assessed any pharmacological (e.g., cannabinoids, gabapentin) or non-pharmacological (e.g., CBT, MI) interventions for treating CUD or reducing problematic use. (3) The study included a comparison group that received placebo, no intervention, standard treatment, or an active comparator. (4) Efficacy outcomes were reported, such as reduced cannabis use, abstinence rates, withdrawal symptoms, treatment retention, or cravings.

**Exclusion criteria:** Studies not published in English or not meeting the study design or outcomes of interest as per the eligibility criteria.

The inclusion of non-diagnosed, heavy, or problematic cannabis users aimed to enhance the representativeness of our study population. Recognizing that some studies might not explicitly report participants as having CUD despite meeting the diagnostic criteria, our approach sought to capture this variability. Documenting quantity/frequency thresholds provided a more consistent measure of severity, while the integration of treatment-seeking behavior and validated screening instruments ensured the identification of clinically significant signs of disordered cannabis use and measurable outcomes for comparison.

The key efficacy outcome measures evaluated across included studies were abstinence, reduction in cannabis use frequency or quantity, withdrawal symptoms, cravings, and treatment retention.

### 2.2. Study Selection

**Figure 1:**
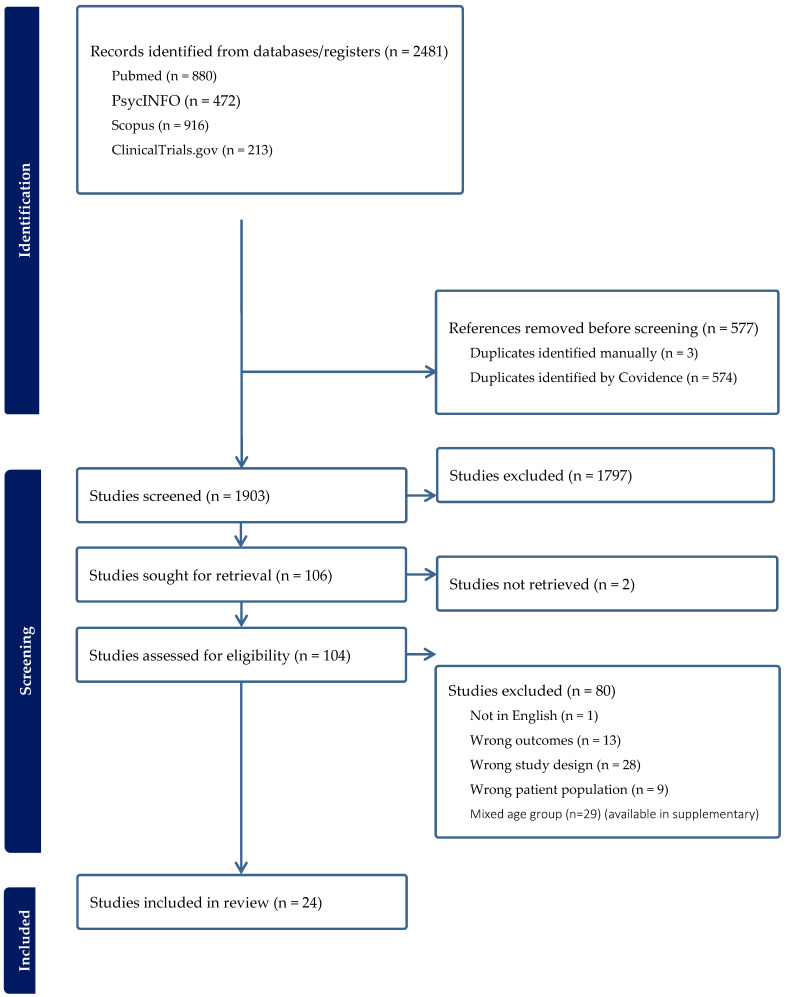
PRISMA 2020 flow chart of literature search.

All records retrieved were imported into Covidence systematic review management software (Veritas Health Innovation, Melbourne, Australia) for screening. After deduplication, two reviewers independently screened titles and abstracts against the eligibility criteria. The full text of any record deemed potentially eligible by either reviewer was retrieved and re-assessed by both reviewers. Disagreements regarding inclusion/exclusion were resolved by consensus. PRISMA standards guided reporting of the study selection process (Figure 1). A standardized data extraction form was created and pilot tested. Two reviewers extracted key data (e.g., study design, sample sizes, demographics, diagnostic criteria, interventions, and outcomes of interest). Any discrepancies in the extracted data were discussed until consensus was reached.

### 2.3. Quality Assessment

Two reviewers independently evaluated risk for included RCTs using the Cochrane risk tool (RoB 2). This tool examines potential issues in five domains: randomization process, deviations from intended interventions, missing outcome data, measurement of the outcome, and selection of the reported result. Each domain was judged as low, high, or some concern. Disagreements were resolved through consensus [39]. RCTs with low risk across all domains were considered low risk overall, and those with some concerns in at least one domain but no high risk were classified as having some concerns overall. The RCTs were deemed high risk if they exhibited high risk in at least one key domain or if there were some concerns in multiple domains in a way that substantially lowered confidence in the reported results. The detailed table assessment is available in the Supplementary File.

### 2.4. Data Synthesis

We stratified participants into three age groups: adolescents (up to 17 years), young adults (18–24 years), and older adults (26–65 years). Studies explicitly targeting adolescents or specific adult age ranges were directly classified based on the delineated target population. For studies where the specific age group was not mentioned, we used the mean and standard deviation (SD) to assign them to the appropriate group [40]. Studies where the age range fell within one SD of a defined age category were classified into that group. This systematic approach enabled reliable classification of studies into the three main age groups for targeted comparison of interventions. The escalation in recent cannabis use among 18- to 25-year-olds emphasized the necessity of separating young adults from older adults in our study [11]. This decision was also supported by relevant literature, aligning with established trends in cannabis use across different adult groups [41,42].

For those studies where age overlapped between different age groups, we classified them as “mixed age group”. Detailed results for these as well as other included studies are provided in the Supplementary File. However, due to the challenge in precisely categorizing their age range, they were not included in the main table. Meta-analysis was not conducted due to substantial heterogeneity across studies in interventions, comparators, and outcome measures.

## 3. Results

We assessed 24 studies examining the effectiveness of interventions targeting cannabis use disorder across three age groups: adolescents (up to 17 years old), young adults (18–24), and older adults (25–65). In the subsequent sections, we will present the notable findings from these studies regarding key outcomes, including abstinence, frequency of use, retention in treatment, and craving/withdrawal, categorized by age group.

### 3.1. Adolescent

Seven controlled trials, which all evaluated non-pharmacological interventions, examined efficacious treatments for cannabis use in adolescents [15,16,17,18,20,21]. The interventions tested included various psychotherapy modalities, contingency management, and brief motivational enhancement.

#### 3.1.1. Abstinence

Two clinical trials showed notable enhancements in cannabis abstinence rates among adolescents with cannabis use disorders. Stanger et al. (2015) conducted a study where participants were randomly assigned to receive either motivational enhancement therapy and cognitive behavioral therapy (MET/CBT), consisting of fourteen 50 min weekly sessions focusing on motivation, self-monitoring, goal setting, coping strategies, and building a support system, or MET/CBT supplemented with a comprehensive contingency management (CM) program. This CM program involved frequent monitoring of urine and breath samples, with rewards provided for negative test results. The study found that adding CM to MET/CBT significantly prolonged abstinence durations compared to MET/CBT alone over a 12-month follow-up period (OR = 1.16, *p* < 0.05) [16].

Mason et al. (2017) took a motivational enhancement approach relying on brief counseling rather than rewards. Their sample was 18 adolescents who used cannabis heavily. The intervention was a 20 min motivational interview session incorporating personalized feedback using a guiding style focused on open-ended questions, reflective listening, and eliciting change talk. The personalized feedback showed where their cannabis use exceeded peer norms. Compared to an attention-control pamphlet review, this brief motivational intervention nearly tripled cannabis abstinence rates at 6-month follow-up (36% vs. 13%, *p* = 0.0034) [18].

#### 3.1.2. Reduced Frequency/Quantity

Lascaux et al. (2016) compared two therapy modalities in adolescents with cannabis dependence—a 6-month formalized therapy program (TAUe) was compared to unstandardized treatment as usual (TAU). The TAUe program incorporated motivational interviewing, experiential techniques, cognitive restructuring, and psychodynamic approaches focused on the adolescent’s substance use. In contrast, the TAU condition had no set format. Results showed that at the 6-month endpoint, adolescents in the TAUe group demonstrated a significantly greater reduction in days of cannabis use per month compared to TAU controls (*p* = 0.032). This difference in favor of the structured therapy remained statistically significant at the 12-month follow-up (*p* = 0.016) [17].

Mason et al. (2017) showed that at the 6-month follow-up, adolescents who received the brief motivational counseling not only had extended periods of abstinence (as previously mentioned) but also a significantly lower probability of using cannabis ≥10 times per month compared to controls (16.6% vs. 38.1% probability, *p* = 0.0034). This demonstrates that even a single abbreviated motivational interviewing session can produce meaningful, sustained effects on reducing frequency of adolescent cannabis use [18].

Lastly, de Gee et al. (2014) examined a motivational enhancement intervention consisting of two 60–90 min individual motivational interviewing sessions spaced one week apart. They compared outcomes to an information control session. Although there was no significant difference between groups on frequency outcomes overall, a subgroup analysis found that heavier users receiving the motivational intervention significantly reduced their quantity of cannabis use over the follow-up period [20].

#### 3.1.3. Retention in Treatment Programs

Kaminer et al. (2017) examined an adaptive treatment program for adolescents with cannabis use disorder. Participants initially received motivational and cognitive behavioral therapy (MET/CBT) for 7 weeks. Those who did not achieve abstinence were categorized as poor responders and randomized to receive an additional 10 weeks of either enhanced CBT or the adolescent community reinforcement approach (ACRA). Only 37% of poor responders completed the full 17-week treatment compared to 78% of those who achieved abstinence with initial MET/CBT (good responders) and required no extra treatment. Additionally, 46% of poor responders failed to complete their assigned extra treatment with enhanced CBT or ACRA versus just 22% non-completion among good responders [15].

Stewart et al. (2015) tested an incentivized intervention by comparing motivational interviewing (MI) alone to MI combined with contingency management (CM), which provided financial rewards for progress towards substance use reduction goals. The incentivized MI + CM group showed increased rates of attending additional treatment sessions after completing the research study compared to non-incentivized MI alone [21].

### 3.2. Young Age Groups

In 11 controlled trials, two assessed pharmacological intervention (vilazodone and topiramate) [30,43], while the others examined non-pharmacological interventions [23,24,25,26,27,28,29,31,32] to determine the most effective interventions for cannabis use in young adults. Below, we outline the significant results related to our outcomes of interest.

#### 3.2.1. Abstinence

One clinical trial found significant improvements in cannabis abstinence rates in young adults with cannabis use disorders. Mason et al. (2018) tested an automated text messaging intervention called Peer Network Counseling-text (PNC-txt) compared to a no-treatment control in young adults with cannabis use disorder. The PNC-txt program delivered personalized text messages over 4 weeks focused on motivation and peer network relationships to achieve abstinence. PNC-txt recipients had significantly higher rates of negative drug screens indicating cannabis abstinence at all follow-ups versus controls. For example, 80% of PNC-txt participants had negative drug screens at the 4-week follow-up compared to 53% of controls [23].

#### 3.2.2. Reduced Frequency and Quantity of Cannabis Use

In Fischer et al. (2016), study participants were randomized to receive either brief oral or written cannabis interventions (C-O or C-W) focused on health risks and motivational strategies to reduce cannabis use, or parallel general health information control conditions (H-O and H-W). Those receiving the combined C-O plus C-W cannabis interventions significantly reduced their number of days of cannabis use over 3 months, lowering use from 23.79 days at baseline to 22.41 days at follow-up [25].

Moving from oral to computerized delivery of interventions, Riggs et al. (2018) evaluated an eCHECKUP marijuana personal feedback program compared to an attention control condition providing general stress management tips. The eCHECKUP group received detailed personalized feedback on their cannabis use frequency, risks for cannabis use disorder, and tips to reduce use. Over 6 weeks, the eCHECKUP group significantly reduced multiple indicators of cannabis use frequency and severity compared to controls, including hours high per week, days high per week, weeks high per month, and weekly use episodes [28].

Transitioning from behavioral to pharmacological interventions, Meisel et al. (2021) examined the opioid antagonist medication topiramate combined with brief motivational and cognitive behavioral therapy (MET-CBT) compared to MET-CBT with placebo. Participants in both groups received only three sessions of MET-CBT, along with either oral topiramate or placebo over 4 weeks. Results found that the topiramate group used significantly fewer grams of cannabis on days they did elect to use over the 6-week trial, exhibiting reduced quantity but not frequency of use. However, the topiramate group did exhibit significantly higher rates of study dropout due to medication side effects [30].

Finally, Macatee et al. (2021) examined an emotional exposure-based distress tolerance training intervention (DTI) compared to a general health education control among young adults with problematic cannabis use and low distress tolerance. Participants received either two 1 h sessions of the computerized DTI program or two 1 h control sessions on sleep/nutrition. Among those with below-average baseline distress tolerance, the DTI group showed significantly greater reductions in their proportion of cannabis use days from pre- to post-treatment, lowering use by 12.2% versus just a 3% reduction in controls [32].

#### 3.2.3. Retention in Treatment

Rigter et al. (2013) compared multidimensional family therapy (MDFT) to individual psychotherapy (IP) for adolescents with cannabis use disorder. MDFT involved two weekly family/parent sessions plus an additional weekly individual session over 5–6 months, focusing on substance use in the family context. Over 12 months, MDFT had significantly higher treatment completion rates than IP (90% vs. 48%) [26].

#### 3.2.4. Cravings

Two trials have demonstrated efficacious interventions for reducing underlying cannabis cravings among young adults. Mason et al. (2018) again examined automated PNC-txt messages versus control and found significantly greater reductions in self-reported craving scores that were sustained over the 3-month follow-up. As previously mentioned, this study also showed the higher abstinence rate following the intervention [23]. 

Also, Meisel et al. (2021) evaluated craving as a secondary outcome in their pharmacotherapy trial combining MET-CBT with topiramate versus placebo. Cravings were significantly blunted in the topiramate group, but dropout due to medication side effects were high in this group [30].

### 3.3. Older Adults

Six clinical trials examined treatment for cannabis use disorder in older adults, with three focusing on pharmacological interventions (lithium, nabiximols, and dronabinol) [33,36,37] and three examining non-pharmacological treatments for this age group. Below, we outline the notable findings from these studies [34,35,38].

#### 3.3.1. Abstinence

Two studies evaluating psychosocial interventions found significant differences in rates of abstinence from cannabis use over time. Walker et al. (2015) compared standard MET/CBT to MET/CBT plus two maintenance check-up (MCU) booster sessions at 1 and 4 months post treatment for adults with cannabis use disorder. Participants received either nine sessions of MET/CBT alone or MET/CBT plus the two MCU boosters. At the 3-month follow-up, those receiving the MCU boosters had significantly higher abstinence rates than standard MET/CBT (36% vs. 13% abstinent, *p* < 0.05). Abstinence rates remained numerically higher with MCU at 9 months [34].

Lintzeris et al. (2020) examined the cannabinoid medicine nabiximols combined with psychosocial interventions compared to placebo plus psychosocial treatment in 128 adults seeking specialized treatment for cannabis dependence. The nabiximols group had significantly higher rates of urine-confirmed abstinence at the 24-week post-treatment follow-up compared to placebo (23% vs. 9%, *p* = 0.035) [36].

#### 3.3.2. Reduced Frequency and Quantity of Cannabis Use

Lintzeris et al. (2020) demonstrated that nabiximols treatment significantly reduced usage days during the 24-week post-treatment follow-up compared to the placebo group (6.7 fewer use days, *p* = 0.006). As previously mentioned, the nabiximols group also had significantly higher rates of urine-confirmed abstinence at follow-up, indicating reduced frequency was associated with increased eventual abstinence [36].

#### 3.3.3. Withdrawal Symptoms

A key challenge in treating adult cannabis use disorder is managing distressing physiological and psychological withdrawal symptoms that frequently lead patients to relapse. Johnston et al. (2014) examined the medication lithium carbonate for managing withdrawal symptoms during cannabis cessation among 38 adult inpatients. Participants underwent a 2-week monitored withdrawal program and were randomized to lithium or placebo for days 2–7. Although lithium did not significantly improve total withdrawal severity scores compared to placebo, it did confer selective relief for the withdrawal symptoms of appetite loss, stomach pain/discomfort, and nightmares. Over half rated lithium as helpful [33].

## 4. Discussion

The use of cannabinoids for recreational and therapeutic purposes has been described for centuries [44]. Cannabis accounts for the third most commonly used substance worldwide, only after alcohol and tobacco [45]. Considering that around 1 in 10 of regular users of cannabis will develop moderate to severe forms of CUD over time, the need for more effective treatments for this condition is pressing [45]. Moreover, the increasing prevalence of cannabis use, CUD, and its complications over the past decade are not negligible, and treatments to date remain insufficient. As of now, there are no FDA-approved medications for this condition, and the off-label use of psychotropic medications has only demonstrated modest to no benefits, particularly for relapse prevention and abstinence [46]. At best, a few psychotropic medications, including cannabinoid agonists, have shown the potential to alleviate cannabis withdrawal symptoms, particularly insomnia, anorexia, and anxiety/restlessness, whereas most placebo-controlled trials for CUD testing a wide range of psychotropic agents have failed to demonstrate benefits for relapse prevention and sustained abstinence [46]. Unfortunately, side effects from these off-label treatments are not insignificant, often limiting compliance and/or resulting in a return to use of cannabis. Moreover, the use of these medications for specific age groups, such as adolescents and older adults, remains unclear, since most studies tend to exclude these specific cohorts from trials. Behavioral and psychosocial interventions remain the main stay treatment for CUD. However, limited access to these evidence-based interventions, engagement, and retention in treatment are often suboptimal, impacting clinical outcomes and overall prognosis.

Notably, although some studies have explored correlates of treatment outcomes, the paucity of data on age-specific treatments for CUD in the existing literature remains. Of note, data suggest that SUDs in older adults remain underestimated and largely untreated, in part because this population has traditionally accounted for only a small fraction of the problem [47,48,49]. The implications of underdiagnosing and undertreating older adults with SUD, including CUD, are particularly concerning, since these individuals are more vulnerable to adverse outcomes associated with drug use [50].

Despite an increasing need for older adult substance use services, facilities with programs designed for older persons remain relatively scarce [51]. In a study of 13,749 responding facilities in the US, only 17.7% had specific programs for older adults [51]. Of note, evidence demonstrates that treatments for SUDs are cost-effective and tend to have similar rates of recurrence/relapse compared to other chronic illnesses in the older adult population [52]. Moreover, evidence has also shown that older individuals tend to have greater adherence to treatment and clinical outcomes, including days of use and abstinence rates regardless of level of care, compared to younger counterparts [53]. Unfortunately, screening, diagnosis, and treatment of SUDs in older individuals remain suboptimal [54,55]. Expanding substance use services, particularly in primary care settings, would likely be considerably impactful, since primary care providers may play an important role in early detection and delivery of brief interventions, particularly for this particular age group [56,57].

This study underscores the imperative of tailoring cannabis use disorder (CUD) treatment according to age, as developmental stages significantly influence the underlying mechanisms of problematic use and the most effective strategies for behavior change. During adolescence, cannabis use often begins as a social activity driven by peer influence and novelty-seeking behaviors [58]. Still-developing cognitive control networks make it difficult for teens to resist immediate rewards and social pressures. Interventions should provide external reinforcement through rewards or environmental modifications to facilitate positive choices. Approaches boosting internal motivation like motivational interviewing also show promise by activating teens’ emerging self-reflective capacities and goal-setting abilities. As supported by the Stanger et al. randomized trial, interventions incorporating external reinforcement such as contingency management with rewards for abstinence can compensate and scaffold improved decision-making during this critical neurodevelopmental window. Specifically, adding a rewards program reliant on frequent drug testing to standard motivational and cognitive behavioral therapy (MET/CBT) prolonged adolescent cannabis abstinence durations over 12 months more successfully than MET/CBT alone [16].

Additionally, even brief counseling approaches exploring internal motivations and self-efficacy show efficacy for this population. The Mason et al. brief motivational interview session focused on eliciting adolescents’ personal reasons for and confidence in abstaining [18]. By emphasizing emergent introspection and metacognitive capacities, this individualized approach nearly tripled cannabis abstinence rates subsequently. This promising efficacy of brief, norms-based feedback on adolescent and youth cannabis outcomes warrants dedicated investigation into real-world implementation systems that could support almost universal reach across educational, workplace, and clinical settings. All youth could be screened for escalating cannabis use and select individuals offered these minimally demanding but high-impact interventions grounded in where their use diverges from peer averages. Preventing and reversing escalation early in critical neurodevelopmental phases can avert years of morbidity and functioning loss—making optimization and dissemination of these interventions an urgent priority with young populations.

Furthermore, for adolescents, involving parents in standard clinic-based treatment significantly improved abstinence outcomes (Stanger 2015), highlighting the vital role of family engagement when targeting youth substance issues [16]. The same emphasis on the role of family engagement was demonstrated in Rigter’s study in young adult age groups, which showed that interventions involving family members significantly impact treatment outcomes [26]. These findings highlight the significance of incorporating family-based interventions into substance use treatment programs for both adolescents and young adults. By recognizing and harnessing the influence of family dynamics, clinicians can enhance treatment effectiveness and promote sustainable recovery outcomes across these age groups.

Although young adults have greater self-regulation capacities, honing emotional and impulse control skills remains a key developmental task. Interventions should enable value exploration to strengthen evolving self-identity [59]. Similar to adolescents, it seems that building distress tolerance and protective peer communities as well as motivational enhancement and coping skill training serves as a fundamental basis for cannabis use interventions in younger adults. For example, a distress tolerance program focused on emotional exposure significantly reduced cannabis use days among young adults prone to distress intolerance (Macatee et al., 2021) [32].

Additionally, the utilization of computerized or mobile programs harnessing technology holds significant appeal for young adults, offering convenient platforms to bolster motivation and facilitate self-monitoring. By integrating young adults’ peer networks, these interventions provide essential accountability and support. For instance, Mason et al. implemented a 4-week automated text messaging program (PNC-txt) aimed at fostering motivation and nurturing positive peer relationships, resulting in notable increases in abstinence rates and reductions in cravings over a 3-month period [23]. Similarly, Riggs et al. demonstrated the effectiveness of a computerized eCHECKUP program, which provided personalized feedback on individual cannabis use patterns alongside tips for reducing use. This intervention yielded significant declines in use frequency and severity [28]. Hence, holistic interventions blending technology-based supports, distress prevention skill building, and positive peer influence optimization demonstrates high promise for reducing cannabis misuse among young adults.

In later life, older adults may face higher challenges, such as chronic pain, sleep disturbances, and mental health issues, that may contribute to relapse into entrenched cannabis use. Unlike other age groups, where studies of pharmacological interventions are limited, there seems to be a notable inclination towards medication-based interventions for older adults, perhaps owing to age-related pharmacokinetic changes and medical comorbidities. For example, Lintzeris et al. found that the cannabinoid medicine nabiximols combined with counseling conferred significantly higher abstinence rates and fewer usage days compared to placebo plus counseling. This underscores the potential utility of pharmaceuticals to help stabilize acute cessation in older individuals by alleviating withdrawal and cravings [36]. Hence, for older adults, an integrative approach blending medication relief from protracted withdrawal with counseling focused on building psychological resilience and social supports appears most effective. Additionally, extended monitoring and booster sessions prove vital for preventing slips from evolving into full relapse across aging. Walker et al. demonstrated that incorporating brief maintenance check-up sessions into standard psychosocial care improved short-term abstinence rates [34].

Furthermore, there appears to be disproportionately less research conducted in older adult populations compared to adolescents and young adults. Only 6 of the 24 studies majorly focused specifically on those aged 26–65 years old. This may be because cannabis use peaks in young adulthood, so there is greater perceived need to target those age groups. However, with increasing rates of older adults using cannabis regularly, more studies on effective treatments tailored to that population are warranted [42].

Additionally, evidence suggests potential gender differences in treatment response that may inform further personalization of interventions. Walukevich-Dienst et al. evaluated an online personalized feedback program aimed at reducing problems related to cannabis use among college students. Although no overall effects on use frequency were found, gender-specific analyses revealed that women receiving the personalized feedback intervention reported significantly fewer cannabis-related problems at one-month follow-up compared to female controls. However, no differences between intervention and control groups were observed for men. This indicates that web-based personalized normative and risk feedback may confer greater benefits for female versus male young adults regarding problematic use indicators. These preliminary findings highlight the need to better understand variables moderating treatment response—such as biological sex, psychosocial characteristics, and mechanisms maintaining use—that can enable further individualization of interventions by gender and other individual factors [29].

In summary, effective treatment for cannabis use disorder (CUD) necessitates an individualized approach that considers the diverse age groups affected, tailoring interventions to address specific developmental challenges and opportunities across the lifespan. Customized interventions, whether leveraging contingent rewards for adolescents, technological platforms for young adults, or pharmacological adjuncts for older individuals, have shown promise in enhancing outcomes. However, the optimal combination and sequencing of age-specific treatments remain uncertain, highlighting the need for further comparative effectiveness research to elucidate the most efficacious approaches. Nonetheless, adopting a personalized, lifespan developmental perspective holds potential to improve upon the modest treatment outcomes observed thus far.

## 5. Conclusions

In conclusion, addressing cannabis use disorder (CUD) demands tailored interventions that acknowledge the unique developmental stages and challenges individuals face across their lifespan. Although interventions such as contingent rewards for adolescents, technology-based platforms for young adults, and pharmacological adjuncts for older individuals show promise, the optimal treatment combination remains uncertain. Further research, particularly comparative effectiveness studies, is imperative to delineate the most efficacious approaches. Although psychosocial interventions offer personalized strategies, age-specific pharmacological recommendations lack adequate evidence. Given the considerable variability among age groups, age-specific treatments need to be further explored.

## Figures and Tables

**Table 1:** Summary of cannabis treatment studies for different age groups.

Study ID	Participants	Intervention Group	Control Group	Duration	Outcomes and Details
**Adolescent (Up to 17)**					
Kaminer 2014 [15]	CUD (DSM)	CBT and VBRT (n = 29)	CBT and rewards (n = 30)	10 w	**Frequency/quantity**: No significant difference between groups in linear change in cannabis use. **Other outcomes**: Self-efficacy and coping response also did not improve during treatment.
Stanger 2015 [16]	CUD (DSM)	MET/CBT + CM (clinical and home based) (n = 153)	MET/CBT (n = 50)	14 w + 12 m F/U	**Abstinence**: MET/CBT + CM had significantly longer periods of abstinence than MET/CBT (OR = 1.16, 95% CI = 1.02, 1.32). **Frequency/quantity**: No significant differences between groups in cannabis use frequency during or after treatment. **Retention**: Retention rates were similar between groups.
Lascaux 2016 [17]	CUD (DSM)	Formalized therapy (TAUe) (n = 38)	Treatment as usual (TAU) (n = 35)	6–12 m	**Frequency/quantity**: At 6 months, the TAUe group had significantly greater reduction in days of cannabis use compared to the TAU group (*p* = 0.032). At 12 months, the difference remained significant (*p* = 0.016).
Mason 2017 [18]	Heavy users	PNC (n = 18)	Control session (n = 28)	6 months	**Abstinence**: At 6 months, the PNC group had a 35.9% probability of cannabis abstinence compared to 13.2% in the control group (*p* < 0.05). **Frequency/quantity**: The PNC group had a 16.6% probability of using cannabis 10 times per month versus 38.1% in the control group (*p* = 0.0034).
Kaminer 2017 [19]	CUD (DSM)	* Enhanced CBT or ACRA (n = 80)	No intervention) (n = 81)	17 w	**Abstinence**: 37% of poor responders completed the adaptive treatment phase; 27% achieved abstinence. No significant difference between the CBT and ACRA groups. **Retention**: At week 17, significantly more poor responders continued drug use (91% vs. 71%) and failed to complete treatment (46% vs. 22%) compared to good responders. * The intervention groups consisted of poor responders to MET/CBT, randomized into enhanced CBT or ACRA.
deGee 2014 [20]	Heavy users	Weed-Check intervention (n = 58)	Information session (n = 61)	3 m	**Frequency/quantity**: Heavier users receiving the Weed-Check reduced their quantity of cannabis use more than heavier users in the control group (mean reduction of 6.1 vs. 3.3 joints per week, *p* = 0.05). No significant differences between groups on other outcomes.
Stewart 2015 [21]	Problematic use	MI + CM (n = 68)	MI alone (n = 68)	8 w+ 16 w F/U	**Frequency/quantity**: The MI + CM group had greater reduction in marijuana use frequency at the end of treatment (Cohen’s d = −0.82) compared to MI alone (Cohen’s d = −0.33), but the differences were not significant at 16 week follow-up. **Retention**: The MI + CM group had lower marijuana-related consequences, higher use of coping strategies, and increased likelihood of attending additional treatment.
**Young Adults (18–24)**					
McRae-Clark 2016 [22]	CUD (DSM)	Vilazodone (n = 41)	Placebo tablets (n = 35)	8 w	**Frequency/quantity**: No significant difference between vilazodone and placebo groups on cannabis use outcomes. Vilazodone did not provide an advantage over placebo in reducing the cannabis use and craving score. **Craving**: Vilazodone did not provide an advantage over placebo in reducing the craving score.
Mason 2018 [23]	CUD (DSM)	PNC-txt (n = 15)	Waitlist control (n = 15)	4 w + 3 m F/U	**Frequency/quantity**: No significant difference in past 30-day cannabis use frequency (*p* > 0.05). **Abstinence**: More PNC-txt participants had negative urine screens for cannabis at follow-up (*p* = 0.03). **Craving**: The PNC-txt group had significantly greater reductions in cravings (*p* < 0.05) compared to controls. **Other outcomes**: The PNC-txt group had significantly greater reductions in cannabis problems (*p* = 0.04) compared to controls.
Wolitzky-Taylor 2022 [24]	CUD (DSM)	AMT (n = 26)	CBT (n = 26)	12 w	**Frequency/quantity**: Non-significant differences between groups in cannabis use outcomes, though AMT showed greater reductions. **Retention**: No significant differences between groups in number of sessions completed or rates of assessment completion. **Other outcomes**: AMT had greater reductions in negative affect (*p* < 0.01) compared to CBT.
Fischer 2013 [25]	Heavy users	Cannabis BI (oral (n = 25) or written (n = 47)	General (oral (n: 25) or written (n: 37))	3 m	**Frequency/quantity**: Decrease in mean number of cannabis use days from 23.79 to 22.41 in total sample (*p* = 0.024). **Other outcome**: Reduced driving after cannabis use from 44.44% to 30.65% in combined intervention groups (*p* = 0.02).
Rigter 2013 [26]	CUD (DSM)	MDFT (n = 212)	IP (n = 238)	12 m	**Abstinence**: 18% MDFT cases had no cannabis use disorder at 12 months vs. 15% IP cases (not significant, *p* > 0.05). **Retention**: 90% MDFT cases vs. 48% IP cases completed therapy (*p* < 0.001). **Frequency/quantity**: Mean number of cannabises use days reduced from 59.8 at baseline to 34.0 at 12 months for MDFT and from 61.5 to 42.3 for IP (not significant, *p* = 0.07).
Mason 2018 [27]	CUD (DSM)	PNC-txt (n = 51)	Assessment only (n = 50)	1 m	**Frequency/quantity**: The PNC-txt group reduced heavy cannabis use days (*p* = 0.005). No significant differences in past 30-day cannabis use overall (*p* > 0.05). **Other outcomes**: The PNC-txt group reduced relationship problems due to cannabis use (*p* = 0.011).
Riggs 2018 [28]	Heavy use	eCheckupToGo (n = 144) *	Stress control (n = 154)	6 w	**Frequency/quantity**: The Marijuana eCHECKUP TO GO group reported decreases in estimated use prevalence/descriptive norms (*p* < 0.01) and decreases in hours/days high per week/month (*p* < 0.05). * Marijuana eCheckupToGo is a kind of personalized feedback.
Walukevich-Dienst [29]	Problematic use	PNF plus additional feedback (n = 102)	PNF-only (n = 102)	~1 m	**Frequency/quantity**: no significant differences between groups on cannabis use frequency. **Other outcomes**: Women in the PFI group reported significantly fewer problems than women in the control group at follow-up assessed by MPS. No significant differences between men in the intervention or control groups.
Meisel 2021 [30]	Problemat use	MET-CBT + topiramate (n = 39)	MET-CBT + placebo (n = 26)	6 w	**Frequency/quantity**: The topiramate group had lower grams of cannabis use on use days (*p* < 0.05) but frequency was not reduced compared to placebo (*p* > 0.05). **Craving**: Cravings were significantly blunted in the topiramate group (*p* < 0.05). **Retention**: Significantly fewer participants (48.72%) completed the study in the topiramate group versus 76.92% in the placebo group.
Bonar 2022 [31]	Heavy users	Motivational interviewing and CBT (n = 76)	Attention-placebo control (n = 73)	8 w + 6 m F/U	**Frequency/quantity**: At 6 months, the intervention group reduced cannabis frequency by 30.1% vs. an increase of 6.8% in the control group (non-significant difference in adjusted model). Reduced cannabis use days by 19.2% in intervention vs. 5.1% reduction for control (non-significant). The only significant difference was a greater reduction in vaping days for the intervention (−43.5%) vs. an increase in the control (+16.7%) group (Cohen’s D = 0.40, *p* = 0.020).
Macatee 2021 [32]	CUD (DSM)	DTI on cannabis (n = 30) *	On health topics (n = 30)	~4 m	**Frequency/quantity**: Reduction in proportion of cannabis use days from pre-treatment to post-treatment: 12.2% in DTI group vs. 3% in HVC group (*p* = 0.02). **Abstinence, Craving, and Retention**: No significant differences between groups on other outcomes. * This method involves psychoeducation and imaginal emotional exposure.
**Older Adults (25–65)**					
Johnston 2014 [33]	CUD (DSM)	Lithium carbonate(n = 16)	placebo (n = 22)	1 w + 3 m F/U	**Frequency/quantity**: Both placebo- and lithium-treated participants showed reduced levels of cannabis use but there was no difference between groups (*p* > 0.05). **Abstinence, Withdrawal, and Retention**: No significant differences between groups in total cannabis withdrawal scale scores, retention rates, rates of completion, or abstinence rates. **Other outcomes**: Lithium significantly reduced individual withdrawal symptoms of loss of appetite, stomach aches, and nightmares/strange dreams.
Walker 2015 [34]	CUD (DSM)	MET/CBT + MCU (n = 37)	Only MET/CBT (n = 37)	9 m	**Frequency/quantity**: MCU used cannabis on fewer days at 3 months (25.52 vs. 50.37 days; *p* < 0.05) but the difference was not significant at 9 months (*p* > 0.05). **Abstinence**: MCU had significantly greater abstinent rates at 3 months (36% vs. 13%; *p* < 0.05) and 9 months (26% vs. 7%; *p* < 0.06).
Fuster 2016 [35]	Heavy users	BNI (n = 59)	No intervention (n = 55)	6 w + 6 m F/U	**Frequency/quantity**: No significant difference in days of marijuana use at 6 weeks (*p* = 0.77) or 6 months (*p* = 0.82) between the BNI group and control. **Other outcome**: No significant difference in SIP-D drug problem scores at 6 weeks (*p* = 0.20) or 6 months (*p* = 0.66).
Lintzeris 2020 [36]	Problematic use	Nabiximols plus PI (n = 61)	Placebo and PI (n = 67)	12 w + 3 m F/U	**Frequency/quantity**: The nabiximols group used cannabis on 6.7 fewer days at 24-week follow-up than the placebo group (*p* = 0.006). **Abstinence**: 23% of the nabiximols group was abstinent at week 24 compared to 9% of the placebo group (OR 3.0, *p* = 0.035).
Levin 2021 [37]	CUD (DSM)	Dronabinol (up to 20 mg) (n = 79)	Placebo (n = 77)	8 w	**Frequency/quantity**: No significant differences in the longitudinal pattern of use over time between treatment groups while adjusted by other covariates. The treatment groups had higher odds of moderate versus heavy cannabis use compared to placebo (*p* < 0.05). No such differences between light versus heavy use (*p* > 0.05).
Heitmann 2021 [38]	CUD (DSM)	Treatment as usual (n = 42) *	Placeb+TAU (n:19) or TAU only (n:17)	6- and 12 m F/U	**Frequency/quantity and craving and relapse**: No significant differences were found between the ABM intervention group and control groups on any of the primary outcomes—substance use, craving, or relapse rates. **Other outcomes**: The groups showed similar reductions in use from baseline to post-treatment but relapse by 6–12-month follow-ups. * This method involves CBT-based outpatient treatment + ABM.

**Abbreviations**: peer network counseling (PNC), affect management treatment (AMT), Marijuana Problems Scale (MPS), psychosocial intervention (PI), brief negotiated interview (BNI), Short Inventory of Problems (SIP-D) for drug problems, cognitive behavioral therapy (CBT), adolescent community reinforcement approach (ACRA), treatment as usual (TAU), motivational enhancement therapy (MET), voucher-based reinforcement therapy (VBRT), motivational interviewing (MI), contingency management (CM), distress tolerance intervention (DTI).

## Data Availability

A list of all studies included, with more details, is provided in the Supplementary Materials. The search strategies utilized for the major databases searched have also been included in the Supplementary Materials. Any additional data related to the systematic review may be requested from the corresponding or first author.

## Supplementary Materials

The following supporting information can be downloaded at: https://www.mdpi.com/article/10.3390/brainsci14030227/s1.

## References

[1] Connor J.P., Stjepanovic D., Le Foll B., Hoch E., Budney A.J., Hall W.D. (2021). Cannabis use and cannabis use disorder. Nat. Rev. Dis. Primers.

[2] Wu L.T., Zhu H., Mannelli P., Swartz M.S. (2017). Prevalence and correlates of treatment utilization among adults with cannabis use disorder in the United States. Drug Alcohol Depend..

[3] Compton W.M., Han B., Jones C.M., Blanco C. (2019). Cannabis use disorders among adults in the United States during a time of increasing use of cannabis. Drug Alcohol Depend..

[4] Giannouli V. (2017). Violence in severe mental illness: Is cognition missing in the associations with ethnicity, cannabis and alcohol?. Australas. Psychiatry.

[5] Dharmawardene V., Menkes D.B. (2017). Violence and self-harm in severe mental illness: Inpatient study of associations with ethnicity, cannabis and alcohol. Australas. Psychiatry.

[6] Carvalho A.F., Stubbs B., Vancampfort D., Kloiber S., Maes M., Firth J., Kurdyak P.A., Stein D.J., Rehm J., Koyanagi A. (2019). Cannabis use and suicide attempts among 86,254 adolescents aged 12-15 years from 21 low- and middle-income countries. Eur. Psychiatry.

[7] Subramaniam G.A., Volkow N.D. (2014). Substance misuse among adolescents: To screen or not to screen?. JAMA Pediatr..

[8] Chen K., Sheth A.J., Elliott D.K., Yeager A. (2004). Prevalence and correlates of past-year substance use, abuse, and dependence in a suburban community sample of high-school students. Addict. Behav..

[9] Baandrup L. (2022). Managing the hazards of cannabis use. Acta Psychiatr. Scand..

[10] Leung J., Chan G.C.K., Hides L., Hall W.D. (2020). What is the prevalence and risk of cannabis use disorders among people who use cannabis? a systematic review and meta-analysis. Addict. Behav..

[11] Chawla D., Yang Y.C., Desrosiers T.A., Westreich D.J., Olshan A.F., Daniels J.L. (2018). Past-month cannabis use among U.S. individuals from 2002-2015: An age-period-cohort analysis. Drug Alcohol Depend..

[12] Lees R., Hines L.A., D’Souza D.C., Stothart G., Di Forti M., Hoch E., Freeman T.P. (2021). Psychosocial and pharmacological treatments for cannabis use disorder and mental health comorbidities: A narrative review. Psychol. Med..

[13] Minerbi A., Hauser W., Fitzcharles M.A. (2019). Medical Cannabis for Older Patients. Drugs Aging.

[14] Page M.J., McKenzie J.E., Bossuyt P.M., Boutron I., Hoffmann T.C., Mulrow C.D., Shamseer L., Tetzlaff J.M., Akl E.A., Brennan S.E. (2021). The PRISMA 2020 statement: An updated guideline for reporting systematic reviews. Rev. Esp. Cardiol. Engl. Ed..

[15] Kaminer Y., Burleson J.A., Burke R., Litt M.D. (2014). The efficacy of contingency management for adolescent cannabis use disorder: A controlled study. Subst. Abus..

[16] Stanger C., Ryan S.R., Scherer E.A., Norton G.E., Budney A.J. (2015). Clinic- and home-based contingency management plus parent training for adolescent cannabis use disorders. J. Am. Acad. Child. Adolesc. Psychiatry.

[17] Lascaux M., Ionescu S., Phan O. (2016). Effectiveness of formalised therapy for adolescents with cannabis dependence: A randomised trial. Drugs Educ. Prev. Policy.

[18] Mason M.J., Sabo R., Zaharakis N.M. (2017). Peer Network Counseling as Brief Treatment for Urban Adolescent Heavy Cannabis Users. J. Stud. Alcohol. Drugs.

[19] Kaminer Y., Ohannessian C.M., Burke R.H. (2017). Adolescents with cannabis use disorders: Adaptive treatment for poor responders. Addict. Behav..

[20] De Gee E.A., Verdurmen J.E., Bransen E., de Jonge J.M., Schippers G.M. (2014). A randomized controlled trial of a brief motivational enhancement for non-treatment-seeking adolescent cannabis users. J. Subst. Abuse Treat..

[21] Stewart D.G., Felleman B.I., Arger C.A. (2015). Effectiveness of Motivational Incentives for Adolescent Marijuana Users in a School-Based Intervention. J. Subst. Abuse Treat..

[22] McRae-Clark A.L., Baker N.L., Gray K.M., Killeen T.K., Wagner A.M., Brady K.T., DeVane C.L., Norton J. (2015). Buspirone treatment of cannabis dependence: A randomized, placebo-controlled trial. Drug Alcohol Depend..

[23] Mason M.J., Zaharakis N.M., Russell M., Childress V. (2018). A pilot trial of text-delivered peer network counseling to treat young adults with cannabis use disorder. J. Subst. Abuse Treat..

[24] Wolitzky-Taylor K., Glasner S., Tanner A., Ghahremani D.G., London E.D. (2022). Targeting maladaptive reactivity to negative affect in emerging adults with cannabis use disorder: A preliminary test and proof of concept. Behav. Res. Ther..

[25] Fischer B., Dawe M., McGuire F., Shuper P.A., Capler R., Bilsker D., Jones W., Taylor B., Rudzinski K., Rehm J. (2013). Feasibility and impact of brief interventions for frequent cannabis users in Canada. J. Subst. Abuse Treat..

[26] Rigter H., Henderson C.E., Pelc I., Tossmann P., Phan O., Hendriks V., Schaub M., Rowe C.L. (2013). Multidimensional family therapy lowers the rate of cannabis dependence in adolescents: A randomised controlled trial in Western European outpatient settings. Drug Alcohol Depend..

[27] Mason M.J., Zaharakis N.M., Moore M., Brown A., Garcia C., Seibers A., Stephens C. (2018). Who responds best to text-delivered cannabis use disorder treatment? A randomized clinical trial with young adults. Psychol. Addict. Behav..

[28] Riggs N.R., Conner B.T., Parnes J.E., Prince M.A., Shillington A.M., George M.W. (2018). Marijuana eCHECKUPTO GO: Effects of a personalized feedback plus protective behavioral strategies intervention for heavy marijuana-using college students. Drug Alcohol Depend..

[29] Walukevich-Dienst K., Neighbors C., Buckner J.D. (2019). Online personalized feedback intervention for cannabis-using college students reduces cannabis-related problems among women. Addict. Behav..

[30] Meisel S.N., Treloar Padovano H., Miranda R. (2021). Combined pharmacotherapy and evidence-based psychosocial Cannabis treatment for youth and selection of cannabis-using friends. Drug Alcohol Depend..

[31] Bonar E.E., Goldstick J.E., Chapman L., Bauermeister J.A., Young S.D., McAfee J., Walton M.A. (2022). A social media intervention for cannabis use among emerging adults: Randomized controlled trial. Drug Alcohol Depend..

[32] Macatee R.J., Albanese B.J., Okey S.A., Afshar K., Carr M., Rosenthal M.Z., Schmidt N.B., Cougle J.R. (2021). Impact of a computerized intervention for high distress intolerance on cannabis use outcomes: A randomized controlled trial. J. Subst. Abuse Treat..

[33] Johnston J., Lintzeris N., Allsop D.J., Suraev A., Booth J., Carson D.S., Helliwell D., Winstock A., McGregor I.S. (2014). Lithium carbonate in the management of cannabis withdrawal: A randomized placebo-controlled trial in an inpatient setting. Psychopharmacology.

[34] Walker D.D., Stephens R.S., Towe S., Banes K., Roffman R. (2015). Maintenance Check-ups Following Treatment for Cannabis Dependence. J. Subst. Abuse Treat..

[35] Fuster D., Cheng D.M., Wang N., Bernstein J.A., Palfai T.P., Alford D.P., Samet J.H., Saitz R. (2016). Brief intervention for daily marijuana users identified by screening in primary care: A subgroup analysis of the ASPIRE randomized clinical trial. Subst. Abus..

[36] Lintzeris N., Mills L., Dunlop A., Copeland J., McGregor I., Bruno R., Kirby A., Montebello M., Hall M., Jefferies M. (2020). Cannabis use in patients 3 months after ceasing nabiximols for the treatment of cannabis dependence: Results from a placebo-controlled randomised trial. Drug Alcohol Depend..

[37] Levin F.R., Mariani J.J., Pavlicova M., Brooks D., Glass A., Mahony A., Nunes E.V., Bisaga A., Dakwar E., Carpenter K.M. (2016). Dronabinol and lofexidine for cannabis use disorder: A randomized, double-blind, placebo-controlled trial. Drug Alcohol Depend..

[38] Heitmann J., van Hemel-Ruiter M.E., Huisman M., Ostafin B.D., Wiers R.W., MacLeod C., DeFuentes-Merillas L., Fledderus M., Markus W., de Jong P.J. (2021). Effectiveness of attentional bias modification training as add-on to regular treatment in alcohol and cannabis use disorder: A multicenter randomized control trial. PLoS ONE.

[39] Sterne J.A.C., Savovic J., Page M.J., Elbers R.G., Blencowe N.S., Boutron I., Cates C.J., Cheng H.Y., Corbett M.S., Eldridge S.M. (2019). RoB 2: A revised tool for assessing risk of bias in randomised trials. BMJ.

[40] Lee D.K., In J., Lee S. (2015). Standard deviation and standard error of the mean. Korean J. Anesthesiol..

[41] Mazhar N.M., Lau F., Van Winssen C., Bajaj N., Hassan T., Munshi T., Groll D. (2016). A Retrospective Hospital Database Analysis on Substance Use-Related Emergency Department Visits in an Ontario University. J. Addict. Med..

[42] Mauro P.M., Carliner H., Brown Q.L., Hasin D.S., Shmulewitz D., Rahim-Juwel R., Sarvet A.L., Wall M.M., Martins S.S. (2018). Age Differences in Daily and Nondaily Cannabis Use in the United States, 2002–2014. J. Stud. Alcohol. Drugs.

[43] McRae-Clark A.L., Baker N.L., Gray K.M., Killeen T., Hartwell K.J., Simonian S.J. (2016). Vilazodone for cannabis dependence: A randomized, controlled pilot trial. Am. J. Addict..

[44] Aggarwal S.K., Carter G.T., Sullivan M.D., ZumBrunnen C., Morrill R., Mayer J.D. (2009). Medicinal use of cannabis in the United States: Historical perspectives, current trends, and future directions. J. Opioid Manag..

[45] Moss H.B., Chen C.M., Yi H.Y. (2012). Measures of substance consumption among substance users, DSM-IV abusers, and those with DSM-IV dependence disorders in a nationally representative sample. J. Stud. Alcohol. Drugs.

[46] Brezing C.A., Levin F.R. (2018). The Current State of Pharmacological Treatments for Cannabis Use Disorder and Withdrawal. Neuropsychopharmacology.

[47] Colliver J.D., Compton W.M., Gfroerer J.C., Condon T. (2006). Projecting drug use among aging baby boomers in 2020. Ann. Epidemiol..

[48] Gfroerer J., Penne M., Pemberton M., Folsom R. (2003). Substance abuse treatment need among older adults in 2020: The impact of the aging baby-boom cohort. Drug Alcohol Depend..

[49] Han B., Gfroerer J.C., Colliver J.D., Penne M.A. (2009). Substance use disorder among older adults in the United States in 2020. Addiction.

[50] Campanelli C.M. (2012). Updated Beers Criteria for Potentially Inappropriate Medication Use in Older Adults: The American Geriatrics Society 2012 Beers Criteria Update Expert Panel. J. Am. Geriatr. Soc..

[51] Schultz S.K., Arndt S., Liesveld J. (2003). Locations of facilities with special programs for older substance abuse clients in the US. Int. J. Geriatr. Psychiatry.

[52] McLellan A.T., Lewis D.C., O’Brien C.P., Kleber H.D. (2000). Drug dependence, a chronic medical illness implications for treatment, insurance, and outcomes evaluation. J. Am. Med. Assoc..

[53] Oslin D.W., Pettinati H., Volpicelli J.R. (2002). Alcoholism treatment adherence: Older age predicts better adherence and drinking outcomes. Am. J. Geriatr. Psychiatry.

[54] Naegle M.A. (2018). Alcohol Use Screening and Assessment for Older Adults. Best. Pract. Nurs. Care Older Adults.

[55] Naegle M.A. (2008). Screening for alcohol use and misuse in older adults: Using the Short Michigan Alcoholism Screening Test—Geriatric Version. Am. J. Nurs..

[56] Babor T.F., Higgins-Biddle J.C., Saunders J.B., Monteiro M.G. (2001). The Alcohol Use Disorders Identification Test Guidelines for Use in Primary Care.

[57] Babor T.F., McRee B.G., Kassebaum P.A., Grimaldi P.L., Ahmed K., Bray J. (2007). Screening, Brief Intervention, and Referral to Treatment (SBIRT). Subst. Abus..

[58] Torrejon-Guirado M.C., Baena-Jimenez M.A., Lima-Serrano M., de Vries H., Mercken L. (2023). The influence of peer’s social networks on adolescent’s cannabis use: A systematic review of longitudinal studies. Front. Psychiatry.

[59] Schreiber L.R., Grant J.E., Odlaug B.L. (2012). Emotion regulation and impulsivity in young adults. J. Psychiatr. Res..

