# Supplementary material for: Treatments for Cannabis Use Disorder across the Lifespan: A Systematic Review
Source: Brain Sci. 2024 Feb 28;14(3):227. doi: 10.3390/brainsci14030227 (PMC10968391; doi:10.3390/brainsci14030227)
Supplement: Supplementary file 1 [file brainsci-14-00227-s001.zip › Appendix A (3).pdf]

Ovid

Database(s): APA PsycInfo 1806 to November Week 3 2023
Search Strategy:

#	Searches	Results
1	exp *Drug Abuse/ and exp *Cannabis/	1673
2	((cannabis or Marijuana) adj3 ("use disorder" or abuse or dependence or addiction)).ti.	829
3	1 or 2	2292
4	exp Drug Therapy/	187656
5	exp Bupropion/	1057
6	exp Cannabidiol/	657
7	exp Fluoxetine/	4026
8	exp Gabapentin/	640
9	exp Motivational Interviewing/	3100
10	exp Lysergic Acid Diethylamide/	1306
11	exp Methylphenidate/	4064
12	exp Mobile Health/	2284
13	exp Naltrexone/	2175
14	exp Psychedelic Assisted Therapy/	83
15	exp Quetiapine/	2019
16	exp Rehabilitation/	61188
17	exp Cognitive Behavior Therapy/	54861
18	exp Transcranial Magnetic Stimulation/	10314
19	exp Contingency Management/	3411
20	((Dronabinol and lofexidine) or acetylcysteine or Bupropion or Cannabidiol or drug or fluoxetine or Gabapentin or intervention or interview or "lysergic acid diethylamide" or Management or medication or methylphenidate or "Mobile health" or Naltrexone or pharmacotherapy or psychedelic or psychedelics or Quetiapine or Rehabilitation or therapy or "transcranial magnetic stimulation" or treatment or Varenicline).ti,ab.	1418534
21	or/4-20	1476374
22	3 and 21	1509
23	limit 22 to (english language and yr="2013 -Current")	762
24	limit 23 to human	733
25	limit 24 to (abstract collection or bibliography or chapter or clarification or "column/opinion" or "comment/reply" or dissertation or editorial or encyclopedia entry or "erratum/correction" or interview or letter or obituary or poetry or review-book or review-media or review-software & other or reviews)	69
26	24 not 25	664

27 (case* adj3 (report* or study or studies)).ti,ab.	176767
28 26 not 27	641
29 limit 28 to "0800 literature review"	40
30 28 not 29	601

<u>ClinicalTrials.Gov</u>

Condition or Disease
Cannabis Use Disorder

Other Terms
(Dronabinol AND lofexidine) OR acetylcysteine OR Bupropion OR Cannabidiol OR drug OR fluoxetine OR Gabapentin OR intervention OR interview OR "lysergic acid diethylamide" OR Management OR medication OR methylphenidate OR "Mobile health"

First posted from 01/01/2013 to 12/04/2023

Condition or Disease
Cannabis Use Disorder

Other Terms
Naltrexone OR pharmacotherapy OR psychedelic OR psychedelics OR Quetiapine OR Rehabilitation OR therapy OR "transcranial magnetic stimulation" OR treatment OR Varenicline

First posted from 01/01/2013 to 12/04/2023

PubMed

((abuse, cannabis[MeSH Terms]) OR (cannabis abuse[MeSH Terms]) OR (cannabis dependence[MeSH Terms]) OR (abuse, marijuana[MeSH Terms]) OR (dependence, marijuana[MeSH Terms]) OR (cannabis use disorder[Title]) OR (cannabis disorder[MeSH Terms]) OR (marijuana use disorder[MeSH Terms]) OR (cannabis abuse[Title]) OR cannabis dependenc*[Title]) OR (marijuana abuse[Title]) OR (marijuana dependenc*[Title]) AND ((Treatment[Title]) OR (Psychotherapy[Title]) OR (Pharmacotherap*[Title]) OR (behavioral therap*[Title]) OR (educational therap*[Title]) OR (medication[Title]) OR (drug[Title]) OR (biological intervention[Title]) OR (Motivational Interview*[Title]) OR (Therap*[Title]) OR (N-acetylcysteine[Title]) OR (acetylcysteine[Title]) OR (Gabapentin[Title]) OR (Bupropion[Title]) OR (Naltrexone[Title]) OR (Contingency Management[Title]) OR (transcranial magnetic stimulation[Title])

OR (Cannabidiol[Title]) OR (Support Group[Title]) OR (Family Therap*[Title]) OR (Vocational Rehabilitation[Title]) OR (fluoxetine[Title]) OR (Electroconvulsive therap*[Title]) OR (Mindfulness-based therap*[Title]) OR (Mobile health app[Title]) OR (Varenicline[Title]) OR (methylphenidate[Title]) OR (modafinil[Title]) OR (Lofexidine[Title]) OR (Buspirone[Title]) OR (Rimonabant[Title]) OR (psychedelic[Title]) OR (psilocybin[Title]) OR (lysergic acid diethylamide[Title]) OR (LSD[Title]) OR (dimethyltryptamine[Title]) OR (ayahuasca[Title]) OR (ibogaine[Title]) OR (mescaline[Title]) OR (ketamine[Title])) NOT (Review[Publication Type] OR Systematic Review[Publication Type] OR German[Language] OR Spanish[Language]) AND (2003/1/1:3000/12/12[pdat])

Scopus

(((TITLE ((cannabis W/3 abuse)) OR TITLE ((marijuana W/3 abuse)) OR TITLE (cannabis W/3 dependen*) OR TITLE ((marijuana W/3 dependen*)) OR TITLE (cannabis W/5 "use disorder") OR TITLE (cannabis W/5 disorder) OR TITLE ("marijuana use disorder"))) AND ((TITLE (treatment) OR TITLE (psychotherapy) OR TITLE (pharmacotherap*) OR TITLE ("therapy") OR TITLE ("behavioral treatment") OR TITLE (medication) OR TITLE (drug) OR TITLE ("biological intervention") OR TITLE (intervention) OR TITLE ("motivational interview*") OR TITLE ("cognitive behavioral therapy") OR TITLE (nacetylcysteine) OR TITLE (acetylcysteine) OR TITLE (psychedelic*) OR TITLE (psilocybin) OR TITLE ("lysergic acid diethylamide") OR TITLE (lsd) OR TITLE (dimethyltryptamine) OR TITLE (ayahuasca) OR TITLE (ibogaine) OR TITLE (mescaline) OR TITLE (ketamine))) AND (PUBYEAR > 2012 AND PUBYEAR < 2024)) AND (LIMIT-TO (DOCTYPE , "ar")) AND (LIMIT-TO (LANGUAGE , "English"))
